# Giant Lipoma of the Thumb Pad in a Confined Space: A Clinical Case

**DOI:** 10.7759/cureus.111622

**Published:** 2026-06-27

**Authors:** Roberto Rodriguez-Ramirez, Alondra Elizabeth Garcia duran, Fernando Isaac Recio España, Guillermo Sergio Dorantes-Millan

**Affiliations:** 1 General Surgery, Dr. Fernando Quiroz Gutiérrez” General Hospital, Institute for Social Security and Services for State Workers (ISSSTE), Mexico City, MEX; 2 Plastic and Reconstructive Surgery, Dr. Fernando Quiroz Gutiérrez” General Hospital, Institute for Social Security and Services for State Workers (ISSSTE), Mexico City, MEX

**Keywords:** giant lipoma resection, hand surgeon, plastic hand surgery, surgical case report, thenar lipoma

## Abstract

Lipomas are among the most common benign soft-tissue tumors; however, their occurrence in the hand is uncommon, and giant lipomas in this location are particularly rare. Due to the limited anatomical space in the hand, these lesions can cause functional impairment and compression symptoms.

We present the case of a 73-year-old woman who presented for evaluation with a six-year history of a mass in the right thenar region that had been progressively increasing in size, accompanied by pain during active thumb movements (flexion, adduction, and opposition), paresis, and decreased pinch strength. Physical examination revealed a soft, mobile mass with mild tenderness on palpation and reduced range of motion in the thumb. Imaging studies revealed a well-defined lipomatous lesion measuring 5 × 3 cm, with no findings suggestive of malignancy. The lipoma was excised via a thenar approach, revealing a well-encapsulated mass located beneath the flexor pollicis brevis muscle, with no involvement of adjacent neurovascular or tendinous structures. A complete resection was performed, and histopathological evaluation confirmed the diagnosis of lipoma. The patient had an uneventful postoperative course, and no complications were observed during follow-up.

Although lipomas are common, their occurrence in the hand is less frequent and can complicate surgical access and hand function. Giant lipomas can cause compression symptoms due to the limited space in the hand, which may lead to sequelae. Imaging studies assist us in preoperative planning and in ruling out malignant neoplasms. Complete surgical excision remains the treatment of choice, yielding good results when adjacent structures are preserved. Giant lipomas of the hand, especially in the thenar region, are rare conditions that can significantly impair hand function. Early detection and appropriate surgical treatment are essential to prevent disease progression and restore hand function.

## Introduction

Lipomas are subcutaneous tumors composed of lobules of adipocytes and are often encapsulated by a thin layer of fibrous tissue. These tumors may be mobile or fixed, may or may not be painful on palpation, and have a soft consistency [1]. A clinical diagnosis can be made because they are visible beneath the skin [1,2]. Although lipomas can present variably in different areas of the body, they may cause complications, such as large size or progressive growth, being located near a joint, or exerting extrinsic compression on adjacent structures. In the current case series, it has been observed that they may present symptoms such as a palpable mass, swelling, pain, and decreased range of motion or hand function; furthermore, they were observed more frequently in men than in women in this study [2,3].

Although not very common, this condition may be associated with disorders such as Madelung's disease, Gardner's syndrome, and multiple hereditary lipomas. In general, there is no apparent preference for lipomas to occur more frequently in men or women, and they typically appear between the ages of 40 and 70. Topographically, lipomas most commonly appear on the trunk and extremities [1,3,4]. Imaging tests are essential for making a diagnosis and planning surgical intervention. An ultrasound can be performed initially, but we can also rely on other imaging studies such as CT and MRI, which allow us to assess the size, depth, density (solid or cystic), and vascularity of the lesion [3].

Giant lipomas of the hand are defined as those larger than 5 cm; they are rare, and surgical treatment is indicated in select cases, particularly when they affect quality of life or are symptomatic [1-3,5]. The aim of this study is to present a case report of a giant lipoma of the hand in the thenar region.

## Case presentation

A 73-year-old female patient presented to the clinic with a sensation of a mass in the right thenar region of six years of evolution, with progressive growth, accompanied by stabbing pain and decreased grip strength, where the abductor pollicis brevis and flexor pollicis brevis muscles were affected, with Tinel and Phalen tests being inconclusive. She denied any history of trauma to that area. Her medical history included arterial hypertension and osteopenia. On physical examination, decreased range of motion of the thumb was observed, as well as the presence of a mass in the thenar region, non-adherent to deep planes, with mild pain upon manipulation, and no color changes with digital pressure (Figure 1).

**Figure 1:**
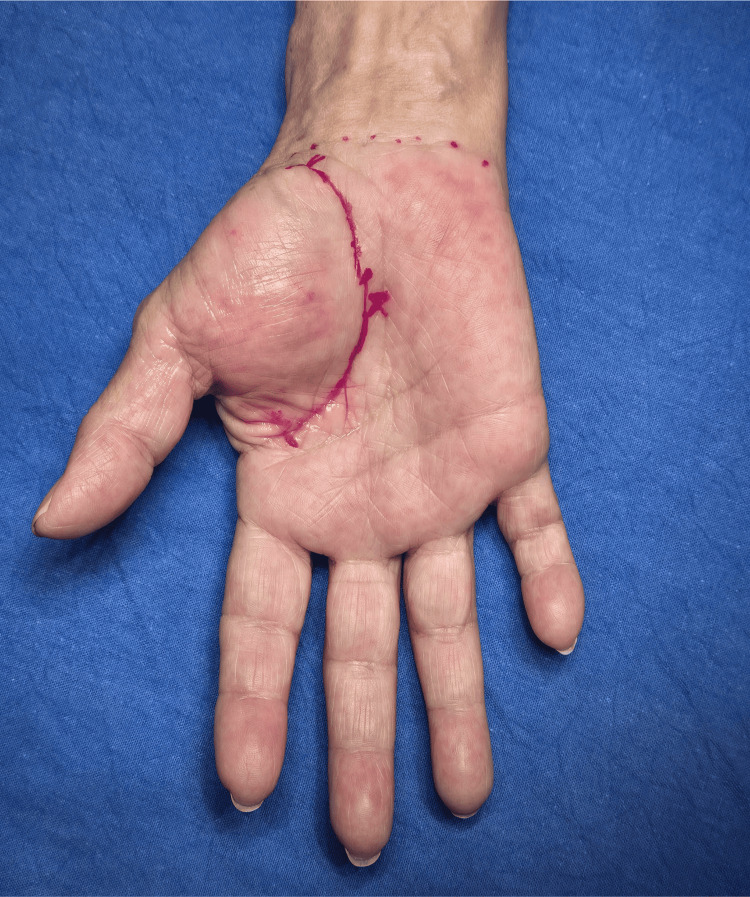
Lipoma in the thenar region Markings are made for a surgical incision on the thenar eminence.

A CT scan of the right upper limb was performed, revealing an ovoid lipomatous lesion in the right thenar region, measuring 51.03 x 33.35 mm, with no contrast enhancement. The appearance was not consistent with a malignant tumor or any type of chronic tenosynovitis (Figure 2).

**Figure 2:**
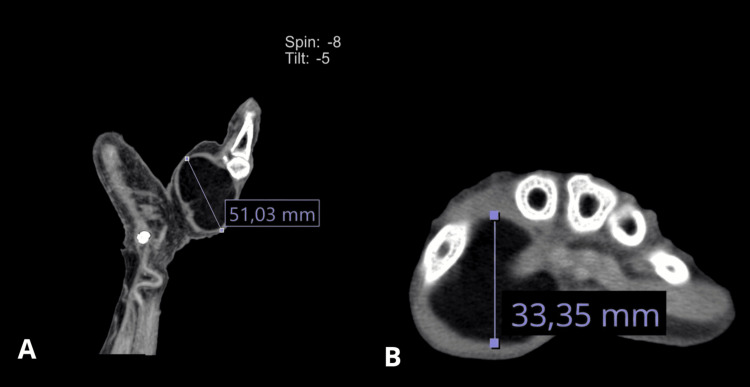
CT of the right hand (A) Sagittal view demonstrating a hypodense soft tissue mass in the thenar region measuring 51.03 mm in its greatest diameter. (B) Axial view showing a hypodense lesion measuring 33.35 mm, consistent with a lipomatous lesion with no evidence of internal calcifications or aggressive features.

Surgical excision of the lipoma was decided. A longitudinal thenar incision was made, revealing a lipoma in the thenar region beneath the flexor pollicis brevis muscle, with no apparent infiltration of the muscle (Figure 3).

**Figure 3:**
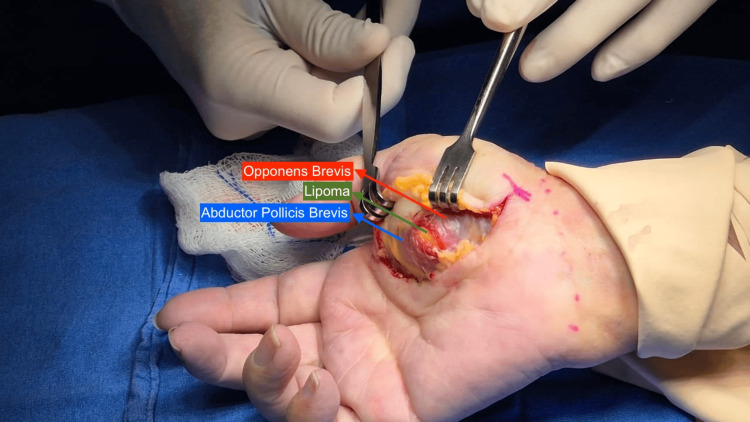
Intraoperative view of the surgical excision of a giant lipoma of the thenar region of the right hand. The encapsulated lipomatous mass (green arrow) is visualized through a palmar incision, with a characteristic yellow-orange lobulated appearance of the lesion.

Dissection was performed, and a 5.1 x 3.3 x 3.2 cm specimen was excised. No nutritional vessel was found, nor were there any adhesions to tendons or adjacent structures. Macroscopically, the specimen exhibited characteristics of a lipoma (Figure 4). It was sent for intraoperative pathological examination, which reported findings consistent with a lipoma. The wound was closed with simple interrupted sutures, and the procedure was concluded.

**Figure 4:**
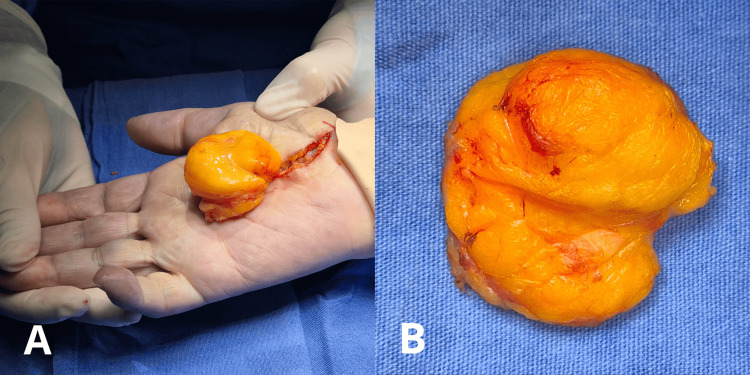
Gross specimen of the resected giant lipoma of the thenar region. (A) Intraoperative view of the excised specimen still partially attached to the surgical site, demonstrating the size of the lesion relative to the patient's hand. (B) A view of the resected specimen on the surgical field.

The patient was discharged that same day with a bandage on her limb, which was kept in place for three days and then removed. She was instructed to perform wound care and passive and active mobilization of her hand at home. She was followed up in the outpatient clinic, where no complications or sequelae were observed.

## Discussion

Lipomas are among the most frequent soft tissue tumors; however, only 1% occur in the hand. These tumors grow within confined spaces and may generate compressive symptoms, particularly within the deep palmar space, which is located behind the flexor tendons and in front of the interosseous and metacarpal muscles. Diagnosis can be established through imaging studies, including ultrasound, CT, and MRI, with histopathological examination performed to confirm the diagnosis [1-6]. Giant lipomas, defined as those exceeding 5 cm, are exceedingly rare and may produce mass effect symptoms as well as extrinsic neurovascular compression and reduced strength or range of motion of the fingers. It has also been documented that they may cause intraosseous nerve compression, carpal tunnel syndrome, and ulnar nerve compression at Guyon's canal [6-7]. In the present case, the patient did not present with neurological symptoms such as paresthesias, atrophy of the thenar muscles, or sensory deficits suggestive of compression of the median or ulnar nerve. This was further corroborated during surgery, during which no invasion of neurovascular structures was observed. The predominant symptoms (pain, decreased grip strength, and limited range of motion of the thumb) were attributable to the mass effect within the anatomical space.

In the present case, no features suggestive of malignancy were identified, such as a firm mass adherent to deep tissue planes or rapid growth. Imaging studies similarly indicated a low probability of malignancy; nevertheless, an intraoperative frozen section study was performed, given the need for histological confirmation that the giant lipoma was of a benign nature.

Surgical treatment is indicated when the tumor begins to exert a mass effect or exceeds 5 cm in its greatest diameter. Furthermore, intramuscular involvement has been identified as a risk factor for liposarcoma [8-9]. In the case of giant lipomas, complete surgical resection is warranted; given that these tumors possess a fibrous capsule, excision may be straightforward provided that the intrinsic nerves of the hand are not involved [9-10].

## Conclusions

Giant lipomas of the hand have a low incidence and should be considered within the differential diagnosis of masses arising in the thenar region. This case highlights that even benign, slow-growing lesions can generate significant compromise of mobility and grip strength due to the limited anatomical space of the hand.

Clinical-radiological correlation is fundamental for guiding the diagnosis and excluding features suggestive of malignancy; however, the definitive diagnosis remains histopathological. Complete surgical resection is not only curative but also allows for adequate functional recovery with a low probability of recurrence.
